# Comprehensive Analysis of CDR3 Sequences in Gluten-Specific T-Cell Receptors Reveals a Dominant R-Motif and Several New Minor Motifs

**DOI:** 10.3389/fimmu.2021.639672

**Published:** 2021-04-13

**Authors:** Shiva Dahal-Koirala, Louise Fremgaard Risnes, Ralf Stefan Neumann, Asbjørn Christophersen, Knut E. A. Lundin, Geir Kjetil Sandve, Shuo-Wang Qiao, Ludvig M. Sollid

**Affiliations:** ^1^ K.G. Jebsen Coeliac Disease Research Centre, Department of Immunology, University of Oslo, Oslo, Norway; ^2^ Department of Immunology, University of Oslo and Oslo University Hospital-Rikshospitalet, Oslo, Norway; ^3^ Department of Gastroenterology, Oslo University Hospital-Rikshospitalet, Oslo, Norway; ^4^ Biomedical Informatics, Department of Informatics, University of Oslo, Oslo, Norway

**Keywords:** celiac disease, T-cell receptors, gluten-specific T-cell receptors, CDR3 motifs, public T-cell receptors, R-motif

## Abstract

Gluten-specific CD4+ T cells are drivers of celiac disease (CeD). Previous studies of gluten-specific T-cell receptor (TCR) repertoires have found public TCRs shared across multiple individuals, biased usage of particular V-genes and conserved CDR3 motifs. The CDR3 motifs within the gluten-specific TCR repertoire, however, have not been systematically investigated. In the current study, we analyzed the largest TCR database of gluten-specific CD4+ T cells studied so far consisting of TCRs of 3122 clonotypes from 63 CeD patients. We established a TCR database from CD4+ T cells isolated with a mix of HLA-DQ2.5:gluten tetramers representing four immunodominant gluten epitopes. In an unbiased fashion we searched by hierarchical clustering for common CDR3 motifs among 2764 clonotypes. We identified multiple CDR3α, CDR3β, and paired CDR3α:CDR3β motif candidates. Among these, a previously known conserved CDR3β R-motif used by TRAV26-1/TRBV7-2 TCRs specific for the DQ2.5-glia-α2 epitope was the most prominent motif. Furthermore, we identified the epitope specificity of altogether 16 new CDR3α:CDR3β motifs by comparing with TCR sequences of 231 T-cell clones with known specificity and TCR sequences of cells sorted with single HLA-DQ2.5:gluten tetramers. We identified 325 public TCRα and TCRβ sequences of which 145, 102 and 78 belonged to TCRα, TCRβ and paired TCRαβ sequences, respectively. While the number of public sequences was depended on the number of clonotypes in each patient, we found that the proportion of public clonotypes from the gluten-specific TCR repertoire of given CeD patients appeared to be stable (median 37%). Taken together, we here demonstrate that the TCR repertoire of CD4+ T cells specific to immunodominant gluten epitopes in CeD is diverse, yet there is clearly biased V-gene usage, presence of public TCRs and existence of conserved motifs of which R-motif is the most prominent.

## Introduction

Celiac disease (CeD) is a prevalent and autoimmune like disorder caused by a maladapted immune response to dietary cereal gluten in genetically predisposed individuals (1, 2). The majority of the patients (~90%) express the HLA-DQ allotype HLA-DQ2.5 (HLA-DQA1*05/HLA-DQB1*02), while the remaining express HLA-DQ2.2 (HLA-DQA1*02/HLA-DQB1*02) or HLA-DQ8 (3). These HLA-DQ molecules present deamidated gluten peptides to CD4+ T cells. On recognition of defined epitopes by their T-cell receptors (TCRs), the T cells become activated, and they then drive a pathogenic immune response by providing help to B cells to differentiate into antibody-producing plasma cells and by communicating with intraepithelial lymphocytes that kill enterocytes (4). Analysis of the TCR repertoires of T cells specific to given immunodominant gluten epitope has revealed V-gene bias and some examples of preferred usage of CDR3 motifs (5–15). The most striking feature has been observed in DQ2.5-glia-α2-specific T cells where dominant usage of TRAV26-1/TRBV7-2 and where many of these TCRs use a CDR3β motif with a conserved non-germline encoded arginine residue (ASSxRxTDTQY, so-called R-motif) and a CDR3α motif (NDYKLS) (5, 7, 11). So far, no comprehensive and comparative analysis of TCR repertoires and CDR3 motif usage by T cells specific for different immunodominant gluten epitopes have not been undertaken.

Here we have performed a systematic search for TCR CDR3 motifs which are prevalent and/or public (i.e. motifs shared by two or more individuals) by analyzing gluten-specific T cells that were isolated using a pool of HLA-DQ2.5:gluten tetramers representing the epitopes (DQ2.5-glia-α1a, DQ2.5-glia-α2, DQ2.5-glia-ω1 and DQ2.5-glia-ω2) from peripheral blood or gut of a collection of HLA-DQ2.5+ CeD patients (11, 12, 16). From sequence analysis of more than 3000 gluten-specific T-cell clonotypes, we are able to describe TRAV and TRBV usage and the global picture of CDR3 motifs carried by T cells specific for these four immunodominant epitopes. The work is important as it lays the foundation for the potential use of gluten-specific TCRs as diagnostic markers of celiac disease.

## Material and Methods

### Generation and Analysis of Single-Cell TCR Sequences From Gluten-Specific CD4+ T Cells

We obtained T cells from a total of 50 HLA-DQ2.5+ patients comprising of patients in active disease state, in remission and patients undergoing gluten challenge recruited to several published/unpublished studies (**Supplementary Table 1**). The studies were approved by Regional Committee for Medical and Health Research Ethics South‐East Norway (REK no. 6544).

The gluten-specific T cells were selected using HLA-DQ2.5:gluten tetramers from peripheral blood mononuclear cells (PBMCs), from lamina propria T cells of gut biopsies, or from *in vitro* cultured T-cell lines (TCLs) as described in previous study (16). Most T cells included in the analysis (82%) were isolated with a cocktail of four immunodominant gluten epitopes DQ2.5-glia-α1a, DQ2.5-glia-α2, DQ2.5-glia-ω1 and DQ2.5-glia-ω2 (**Supplementary Table 2**). Single cell suspension generated from lamina propria of gut biopsies were stained with PE‐conjugated HLA-DQ2.5:gluten tetramer/s (10 µg/mL) for 30–45 min at room temperature before adding antibody mixtures reactive with cell surface markers. Live, single, CD3+, CD11c−, CD14−, CD15−, CD19−, CD56−, CD8−, CD4+, CD8- and HLA‐DQ2.5:gluten tetramer+ cells were then isolated by FACS. PBMCs were stained with PE‐conjugated HLA-DQ2.5:gluten tetramer/s (10 µg/mL) for 30–45 min at room temperature prior to magnetic bead enrichment of tetramer binding cells followed by antibody staining. Live, single, CD3+, CD11c−, CD14−, CD15−, CD19−, CD56−, CD4+, CD45RA−, CD62L−, integrin β7+, and HLA‐DQ2.5:gluten tetramer+ were sorted for TCR sequencing. TCLs were stained with PE conjugated HLA‐DQ2.5:gluten tetramer (10 µg/mL) for 2 h at 37°C prior to adding antibody cocktail. We sorted live, single, CD3+, CD8−, CD4+ and HLA‐DQ2.5:gluten tetramer+ cells. The following antibodies were used in the study: CD14‐Pacific Blue (Biolegend), CD15‐Pacific Blue (Biolegend), CD19‐Pacific Blue (Biolegend), CD56‐Pacific Blue (Biolegend), CD3‐FITC (Biolegend) or CD3‐Superbright 600 (eBioscience) or CD3‐ Brilliant Violet 510 (Biolegend), CD11c‐Horizon V450 (BD Biosciences), CD4‐APC‐H7 (BD Biosciences), CD62L‐PerCP/Cy5.5 (BD Biosciences), CD45RA‐PECy7 (eBioscience), integrin‐β7‐APC (Biolegend), and CD8‐PerCP (eBioscience). To exclude dead cells we used LIVE/DEAD marker fixable violet stain (Thermo Fischer Invitrogen). The FACS plot showing gating strategy for the isolation of T cells from blood, gut and TCLs is shown in **Supplementary Figure 1**.

Single T cells were sorted on 96-well plates and rearranged TCRα genes and TCRβ genes were amplified by multiplex PCR with V-gene specific primers described in detail elsewhere (16). Sequencing was performed on the Illumina MiSeq platform (250 bp PE) at the Norwegian Sequencing Center (Oslo University Hospital). The single‐cell TCR‐αβ sequencing raw data generated are available in European Genome‐phenome Archive (EGAS00001003245) and NCBI’s Sequence Read Archive database (SRP102399 and SRP102402) (12, 16), or as new deposits (EGAS00001005047) (**Supplementary Table 1**). A compilation of TCR nucleotide and amino acid sequences of 2918 clonotypes are available from the authors on reasonable request.

Raw Illumina sequencing reads were assembled into rearranged TCR sequences using the MiXCR (17) “analyze amplicon” macro (default settings). Subsequently, for each cell the three highest scorings in terms of read support, TCRα and TCRβ MiXCR sequences were submitted to the IMGT HighV-QUEST web portal (18), resulting in TCR annotation output that is compatible with our in-house Immune Receptor Information System (IRIS). IRIS is a data repository as well as a data analysis software. We upload the IMGT output files into the IRIS database followed by connecting the data with the metadata by assigning different dimensions (age, patient ID, status, tissue, well ID, plate ID, library ID, etc.) to each of the sequences. This allows the user to choose any specific dataset based on these dimensions. Further filtering steps were carried out in the IRIS program in order to remove low quality or ambiguous sequences. Sequences with a read support of <50 were discarded. Valid cells were defined as comprising of one or two TCRα and TCRβ sequences, maximum three sequences. Dual TCRα or TCRβ were accepted based on read support proportions. The number and frequency of dual productive TCRα (median:6.9%) or TCRβ (median:0.2%) and unproductive TCRα (median:16.1%) or TCRβ (median:3.5%) in each patient is shown in **Supplementary Table 1** and **Supplementary Figure 2**. After the initial filter has been set up, IRIS will perform downstream analysis on the data set requested by the user on only the sequences that pass through the filter. So, using IRIS, users can choose specific data based on the dimensions, filter the sequences and the sequences can then be browsed directly, or used as the input when creating a report on V-gene usage, pairing and other relevant TCR analysis. Previously, we used pRESTO to process the raw sequencing reads (16), but here we have used MiXCR as it improved the processing pipeline and also increased the number of valid cells by roughly 10%. Clonotypes were defined based on identical TCRβ sequences (identical V (gene level) - and J genes and identical CDR3 nucleotide sequences); subsequent inspection confirmed that more than 99% of clonotypes were characterized by unique TCRα:TCRβ combinations. In total, 6627 valid cells from 50 patients gave rise to a total of 2918 clonotypes, ranging from 201 to 1 clonotypes per patient (**Supplementary Table 1**). Only the 34 patients with at least 20 clonotypes (2764 clonotypes in total) were included in the clustering and motif discovery analyses (**Supplementary Table 1**). The remaining 16 patients with less than 20 clonotypes per patient were included in assessing sharing of TCR sequences across patients.

V-gene usage for the single-cell sequencing libraries was exported from IRIS; here, sequences with ambiguous V gene calls were removed and the distinct V gene were counted. For paired TCR V-gene usage, the combinations of TRAV and TRBV genes were counted. In case of dual TCRα or TCRβ, both TRAV and TRBV gene combinations were included and each was counted 50%.

### Hierarchical Clustering

For the hierarchical clustering we removed from the CDR3 sequences two amino acids from the N terminal (IMGT positions 105 and 106) and one amino acid from the C terminal (IMGT position 117) following the recommendation of Glanville et al. These authors demonstrated based on assessment of 52 ternary TCR-peptide-MHC structures that these TCR residues do not contact peptide antigen thereby leading to the recommendation that clustering analysis for shared specificity should be performed without these residues (19). Additionally, we removed all TCR sequences where the length of the remaining CDR3 was less than 8 amino acids. For paired TCR sequences with dual TCRα or TCRβ, one of the dual sequences was selected at random to represent the TCR. These operations reduced the size of the dataset from 2764 clonotypes to 2750 clonotypes.

Hierarchical clustering was done using the python “scipy.cluster.hierarchy” package, selecting average (UPGMA) linkage. Pairwise distances between amino acid TCRα or TCRβ CDR3s were calculated using the Levenshtein editing distance, using a value of 0 for matching positions and 1 for mismatches and indels. Pairwise distances between paired TCRα:TCRβ receptors were calculated as the sum of the TCRα and TCRβ Levenshtein distances.

### Motif Discovery

New motif candidates were retrieved by examining all nodes of the hierarchical clustering dendrogram in a recursive, root-to-leaves ordering. Nodes below a given Levenshtein cut-off distance were assessed in terms of the V genes utilized by their leaf clonotype sequences. If all leaf clonotypes of a given node were utilizing similar V genes, and at least five leaf clonotypes were present, a new motif candidate was assigned to this node. In this case, the recursive search would stop. The required similarity of V genes was either defined as identical V genes (disregarding the allele numbers), or alternatively identical V-gene subgroups (as used by IMGT). Resulting motifs matching any of the established CeD motifs (**Table 1**) were removed from the list. For each remaining motif, a multiple sequence alignment was created using ClustalW (20); corresponding sequence logos were created using the WebLogo tool (21). In case of identical characters for a given column, the column character was added to the regex. If several distinct characters belonging to the same amino acid group; charged (K, R, E, D), polar (Q, N, H, S, T, Y, C), amphipathic (W, Y, M), hydrophobic (A, I, L, M, F, V, P, G) were present, a character group containing all amino acid residues for that group were added to the regex. If more than one amino acid group was represented, the position was added as an unrestricted wild-type character. If a column contains one or more gap characters, the amino acid character(s) for that position were marked as optional. Finally, all regex search terms were associated with the V gene used by the motif. Sequences constituting matches to a certain motif were required to match both the motif regex and express identical V gene. For paired TCR sequences, matches for both TCRα and TCRβ had to be found.

**Table 1:** CeD-relevant sequence motifs.

Motif name	Chain	Sequence	V gene	J gene	Pairs with	Frequency
R-motif	TCRβ	ASS.R.TDTQY	TRBV7-2 or TRBV7-3	TRBJ2-3	TRAV26-1 (87.6%)	291/2764 (10.5%)
					TRAV13-1 (1.3%)	
					TRAV23/DV6 (1.3%)	
					Others (9.8%)	
Extended R-motif	TCRβ	ASS.R.*	TRBV7-2 or TRBV7-3	TRBJ2-3	TRAV26-1 (81.1%)	369/2764 (13.3%)
					TRAV14/DV4 (2.0%)	
					TRAV13-1 (1.8%)	
					TRAV23/DV6 (1.5%)	
					TRAV4 (1.3%)	
					Others (12.3%)	
NDYKLS	TCRα	I.NDYKLS	TRAV26-1	TRAJ20	TRBV7-2 (88.6%)^$^	43/2764 (1.6%)
					TRBV20-1 (4.6%)	
					TRBV11-2 (2.3%)	
					TRBV5-1 (2.3%)	
					TRBV7-3 (2.3%)	
Paired R-motif	TCRαβ	ASS.R.TDTQY	TRBV7-2 or TRBV7-3/TRAV26-1	TRBJ2-3	N/A	

^$^All of these TCRβ sequences contain the R-motif.

The sequence patterns are displayed using regular expression syntax where “.” Indicates any character and “.*” indicates any characters including no character.

### Linking Motifs to Epitope Specificity

In order to evaluate the epitope specificity of the new motifs obtained by hierarchical clustering, we looked into the TCRs of *in vitro* cultured gluten-specific T-cell clones (TCCs) with known epitope specificity. Over the years, we have generated TCCs reactive to different gluten epitopes from blood and gut samples obtained from 15 CeD patients of whom 13 are not included in the clustering analysis. The TCR sequences and relevant patient information for the TCCs used in this study is shown in **Supplementary Table 3**.The majority of the TCCs were generated from CD4 T cells sorted with HLA-DQ2.5:gluten tetramers by limited dilution cloning and antigen‐free expansion. However, some of the TCCs were generated by cloning of T-cell lines generated from biopsies of CeD patients and the epitope specificity was confirmed by T-cell proliferation assays. Of the 328 gluten-specific TCCs, 231 TCCs expressed unique TCRs (the remaining 97 TCCs were sister clones) (**Supplementary Table 3**). We had 39, 111, 13 and 54 unique TCCs specific for DQ2.5-glia-α1a, DQ2.5-glia-α2, DQ2.5-glia-ω1, and DQ2.5-glia-ω2, respectively. Further, we also had 10 and 6 unique TCCs that were cross-reactive for DQ2.5-glia-α1a and DQ2.5-glia-ω1 epitopes, or DQ2.5-glia-α2 and DQ2.5-glia-ω2 epitopes, respectively.

### Search of CDR3 Motifs in Public TCR Databases

We searched for all the CDR3 motifs in two different TCR databases, VDJdb repository (https://github.com/antigenomics/vdjdb-db/releases/tag/2020-01-20) and McPAS-TCR (22). These databases contain TCRs with specificity for known epitopes. TCR specific for CeD related epitopes were excluded prior to the search. Sequences matching a certain motif were required to match both the motif regex and possess identical V gene.

### Immune-Receptor Generation Probability (IRGP) Calculations

The IGoR (23) and OLGA (24) programs were used for calculating IRGPs. For sequences with ambiguous V gene calls, one of the V gene possibilities were chosen at random to represent the sequence in question. Since sequencing reads were not long enough to allow confident allele identifications, the “*01” allele was assumed used for all V genes. IRGPs were calculated using a default IGOR installation, together with the default TCRα and TCRβ models included in (23). IRGPs for paired TCRαβ sequences (amino acid and nucleotide level) were calculated as the products of the TCRα and TCRβ IRGPs (25).

## Results

### TRAV, TRBV and TRAV : TRBV Usage by Gluten-Specific TCRs in CeD

We analyzed the V genes used by gluten-specific T cells of 31 CeD patients. The T cells embodying altogether 2396 clonotypes were isolated using a cocktail of HLA-DQ2.5:gluten tetramers representing four immunodominant gluten epitopes (DQ2.5-glia-α1a, DQ2.5-glia-α2, DQ2.5-glia-ω1 and DQ2.5-glia-ω2). For this analysis we excluded 20 patients where we isolated T cells using HLA-DQ2.5:gluten tetramers representing only one or two epitopes to avoid potential epitope-specific V-gene bias.

**Figure 1:**
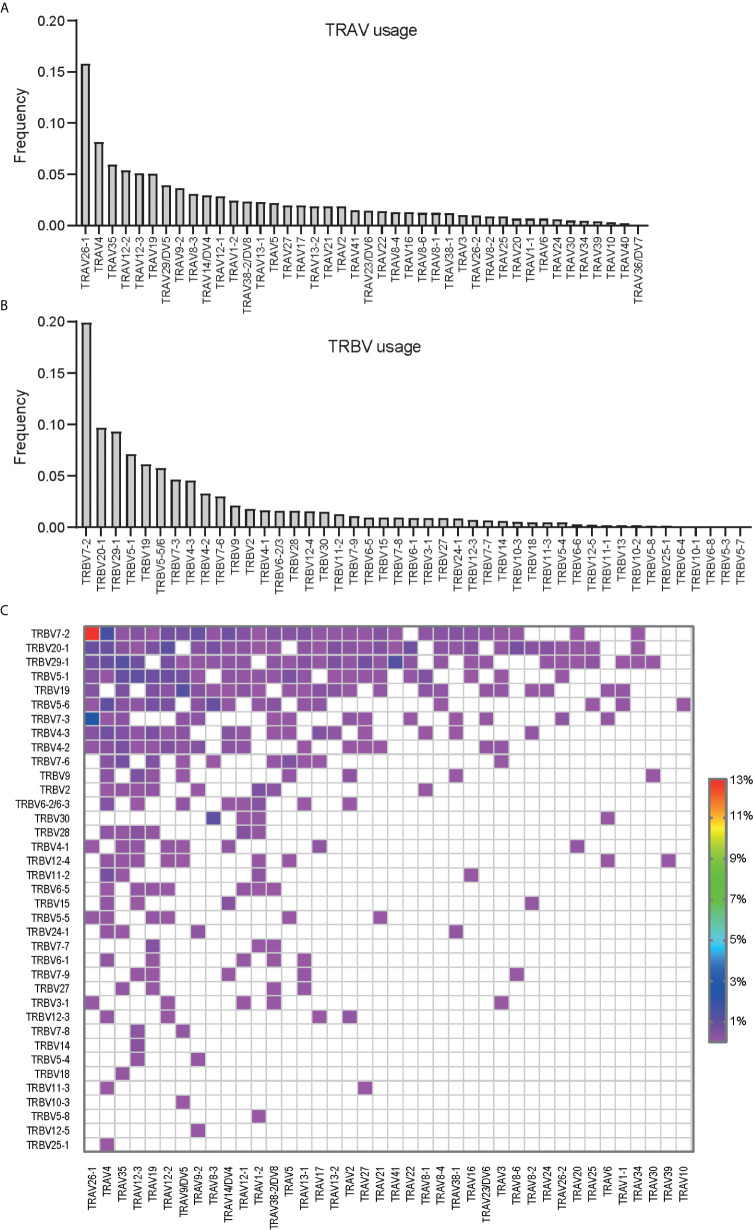
The **(A)** TRAV, **(B)** TRBV and **(C)** paired TRAV : TRBV gene usage in T cells specific to immunodominant gluten epitopes isolated using a cocktail of HLA-DQ2.5:gluten tetramers representing four immunodominant gluten epitopes (DQ2.5-glia-α1a, DQ2.5-glia-α2, DQ2.5-glia-ω1 and DQ2.5-glia-ω2). In total 2396 clonotypes from 30 CeD patients were analyzed where the V genes or V gene pair expressed in at least two clonotypes were included.

TRAV26-1, TRBV7-2 and TRAV26-1:TRBV7-2 were found to be the most prominently used V genes and V gene pair used by the gluten-specific T cells (**Figure 1**). The analysis revealed the biased expression of V genes that previously were described to be used by TCRs specific to one of these immunodominant epitopes such as TRAV26-1, TRAV4, TRAV35, TRAV12-2/3 for TRAV (**Figure 1A**), and TRBV7-2, TRBV29-1, TRBV20-1, TRBV5-1 and TRBV19 for TRBV (**Figure 1B**). Except for TRAV26-1:TRBV7-2 which was expressed in 12% of the clonotypes, the majority of the V gene pairs were expressed at low frequency (< 1%) indicating that the gluten-specific TCRs against these four immunodominant gluten epitopes express diverse TCRs with the exception of TRAV26-1:TRBV7-2 TCRs (**Figure 1C**). This suggested that the biased expression of TRAV26-1:TRBV7-2 is the most prominent feature of TCRs specific to HLA-DQ2.5-restricted immunodominant gluten epitopes in CeD.

### Hierarchical Clustering on CDR3 Sequences Places the R Motif as the Most Prominent CeD Relevant Motif

In order to reveal TCR motifs associated with CeD, we performed hierarchical clustering on CDR3 amino acid sequences derived from 34 patients that had more than 20 clonotypes (2750 clonotypes in total). All clonotypes recognized one or more of the DQ2.5-glia-α1a, DQ2.5-glia-α2, DQ2.5-glia-ω1 and DQ2.5-glia-ω2 epitopes presented on HLA-DQ2.5 (**Supplementary Table 2**). Clustering was done based both on the individual CDR3α and CDR3β sequences, and of paired TCRαβ receptors represented by their CDR3 sequences. Further, the pairwise distances between sequences were calculated using the Levenshtein editing distance.

**Figure 2:**
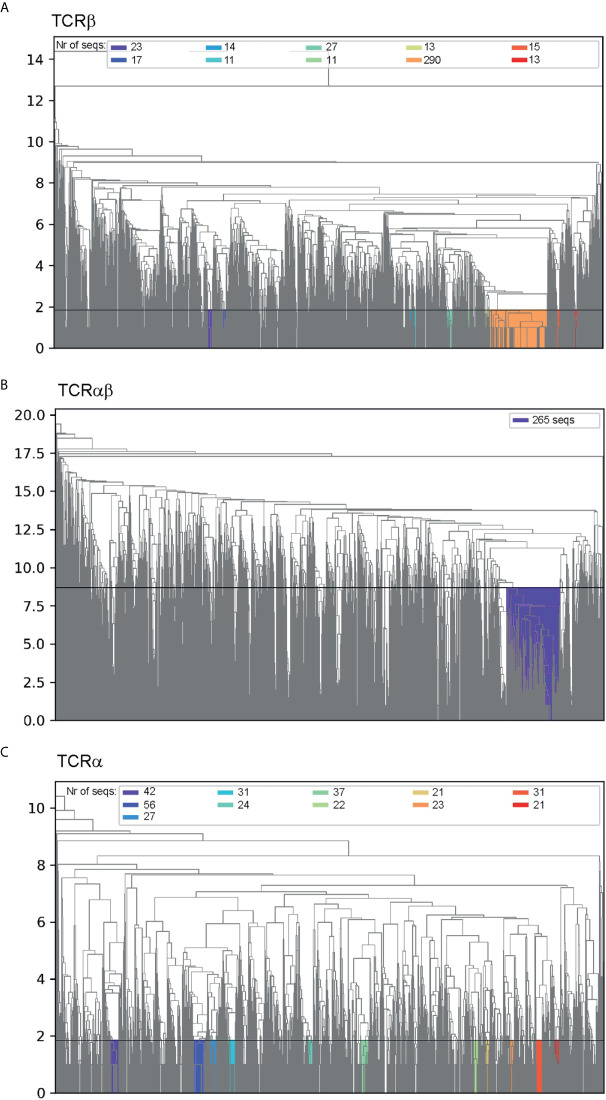
Hierarchical clustering of **(A)** CDR3β sequences, **(B)** paired CDR3α:CDR3β sequences and **(C)** CDR3α sequences. The y-axis indicates the Levenshtein distance and the horizontal line shows distance that was used to cut the dendogram tree to generate the clusters. The clusters are colored and the respective number of sequences in each cluster are indicated in the legend.

The clustering dendrograms for CDR3β sequences revealed that the largest cluster (**Figure 2A**) observed at a level of average Levenshtein distance of ~1.85 contained R-motif (**Table 1**) clonotypes in the dataset, and only 1.7% of the cluster clonotypes did not match this motif. Comparable clusters obtained by cutting the dendrogram tree at the same Levenshtein distance were considerably smaller, where the largest of which contained 27 clonotypes compared with 290 clonotype for the R-motif cluster.

Similarly, the largest cluster observed in clustering dendrograms for paired CDR3α:CDR3β sequences at a Levenshtein cut-off distance of 8.7 comprised mostly (85.3%) of paired R-motif (**Table 1**) and R-motif for TCRβs (91.1%) (**Figure 2B**). The relatively high Levenshtein editing distance of 8.7 compared to the TCRβ motif (**Figure 2A**) reflects the heterogeneity of the CDR3α sequences. However, if the dendrogram tree was cut at a distance of 2.4, the R-motif paired to the NDYKLS-motif was found to be the largest cluster (39 clonotypes) at that distance (**Supplementary Figure 3**).

Clustering the CDR3α sequences in the same manner as for CDR3β sequences above produced a dendrogram that did not contain any distinct clusters. However, at the cut-off distance of 1.85 (**Figure 2C**), we identified a cluster containing the NDYKLS-motif, with 56 sequences. This cluster was the largest CDR3α cluster at this cut-off distance as the other clusters contained 21 to 42 sequences.

Taken together, hierarchical clustering on CDR3 sequences revealed that the well-known R motif is by far the most prominent CeD relevant motif.

### Hierarchical Clustering on CDR3 Sequences Reveals New Smaller Motifs

In order to identify potential new motifs, we looked for clusters of CDR3 sequences within a given Levenshtein distance with the additional requirement that all clonotypes within such a cluster should use the same V gene.

Performing a motif search using the CDR3α based clustering, 38 and 37 new CDR3α motif candidates for Levenshtein distance 1.0 (**Supplementary Figure 4A**) and 2.0 (**Supplementary Figure 4B, C, Supplementary Table 4**), were identified. For both distances, the largest new motif was about equal in size to the NDYKLS-motif.

Based on the CDR3β clustering, 25 and 32 new motif candidates were identified for Levenshtein distances of 1.0 (**Supplementary Figure 5**) and 2.0 (**Supplementary Figure 5B, C, Supplementary Table 5**). For both Levenshtein distances, the resulting new motif clusters were all substantially smaller than the R-motif cluster, which was represented with 290 sequences in the dataset.

Finally, we identified new motif candidates based on clustering of the CDR3α:CDRβ sequences, requiring identical V genes for both TCRα and TCRβ sequences. As we included both sequences, we doubled the allowed Levenshtein distances to 2.0 (**Supplementary Figure 6A**) and 4.0 (**Supplementary Figure 6B, C, Supplementary Table 6**). This yielded 18 and 27 new motif candidates, respectively. For a cut-off distance of 4.0, the largest new motif contained 16 sequences, substantially smaller than the number of receptors utilizing the paired R-motif (275). We also retrieved paired motifs based on identical V gene subgroups and increased the Levenshtein distance cut-off to 8.0 which resulted in 37 new motif candidates. Despite the increased Levenshtein distance, the largest new motif candidate still contained far less clonotypes than the paired R-motif cluster.

Taken together, we identified new CDR3 motif candidates used by the gluten-specific TCRs other that the R-motif. However, the occurrence of these novel motifs were much lower than the R-motif.

### CDR3 Motifs Used by the Gluten-Specific TCRs in Public TCR Databases

As it would be of interest to learn whether the CDR3 motifs of the gluten-specific TCRs are unique to celiac disease, we searched the VDJdb and McPAS-TCR sequence repositories for presence of the motifs. Very few matches were found (**Table 2**). As expected, CDR3 motifs based on alignments with larger editing distances gave more matches. In addition, CDR3α motifs gave more matches than CDR3β motifs. No matches were obtained when searching with paired CDR3α:CDR3β motifs. Likewise, the R-motif and the NDYKLS-motif did not match any sequences. Of the gluten-specific TCRs, only 2.2%, 0.2% and 0% of the CDR3α, CDR3β and paired CDR3α:CDR3β motifs respectively matched sequences in the VDJdb repository. Similarly, the motifs that matched sequences in McPAS-TCR were 0.9%, 0.1% and 0%. These results may indicate that the novel CDR3 motifs, specially the CDR3β and the paired CDR3α:CDR3β motifs are fairly specific to CeD.

**Table 2:** The numbers of sequences from the VDJdb and McPAS-TCR that match the CDR3 motifs of gluten-specific TCRs.

	VDJdb (n=67689)			McPAS-TCR (n=34795)		
	CDR3α (n=28268)	CDR3β (n=39405)	CDR3α:CDR3β (n=22284)	CDR3α (n=11675)	CDR3β (n=13332)	CDR3α:CDR3β (n=7501)
R-motif	–	0	–	–	0	–
Paired R-motif	–	–	0	–	–	0
NDYKLS-motif	0	–	–	0	–	–
Motifs at distance=1	4^a^	1^c^	–	21^a^	3^h^	–
Motifs at distance=2	10^b^	4^d^	0^e^	21^g^	3^i^	0^e^
Motifs at distance=4	–	–	0^f^	–	–	0^f^

^a^Sequence match with 2 out of 38 CDR3α motifs.

^b^Sequence match with 5 out of 37 CDR3α motifs.

^c^Sequence match with 1 out of 25 CDR3β motifs.

^d^Sequence match with 4 out of 32 CDR3β motifs.

^e^Sequence match with 0 out of 18 CDR3α:CDR3β motifs.

^f^Sequence match with 0 out of 27 CDR3α:CDR3β motifs.

^g^Sequence match with 2 out of 37 CDR3α motifs.

^h^Sequence match with 0 out of 25 CDR3β motifs.

^i^Sequence match with 0 out of 32 CDR3β motifs.

### Linking Epitope Specificity to the Motifs Identified by Hierarchical Clustering

We wanted to pinpoint the epitope specificity of obtained novel motifs by comparing with the TCRs of the TCCs with known gluten epitope specificities (**Supplementary Table 3**) as well as single HLA-DQ2.5:gluten tetramers. We also looked for these motifs in the entire dataset comprising of single cell TCR sequencing data from 50 patients and TCR sequences from TCCs from 15 patients (**Supplementary Table 3**) and reported the prevalence in **Supplementary Tables S4-S6**. We found that 84% (31 of 37) CDR3α motifs (**Supplementary Table S4**) and 63% (20 of 32) CDR3β motifs (**Supplementary Table S5**) identified at Levenshtein distance of 4.0, could be linked to TCRs with known specificity. We found that out of 27 paired CDR3α:CDR3β motifs identified at a Levenshtein distance of 4.0, nine motifs were used by at least one gluten-specific TCC (**Supplementary Table 6**). Additional six motifs were linked to TCRs sequenced from T cells that had been isolated with single HLA-DQ2.5:gluten epitope tetramers. Further, three motifs were identified in TCRs of TCCs as well as T cells isolated with single HLA-DQ2.5:gluten epitope tetramers. As a result, we were able to identify 16 new paired CDR3α:CDR3β motifs for DQ2.5-glia-α1a (7), DQ2.5-glia-α2 (1), DQ2.5-glia-ω1 (1), DQ2.5-glia-ω2 (3) and DQ2.5-glia-α1a/DQ2.5-glia-ω1 (4).

### Higher Number of Shared TCR Sequences Observed in Patients With Higher Number of Clonotypes

**Figure 3:**
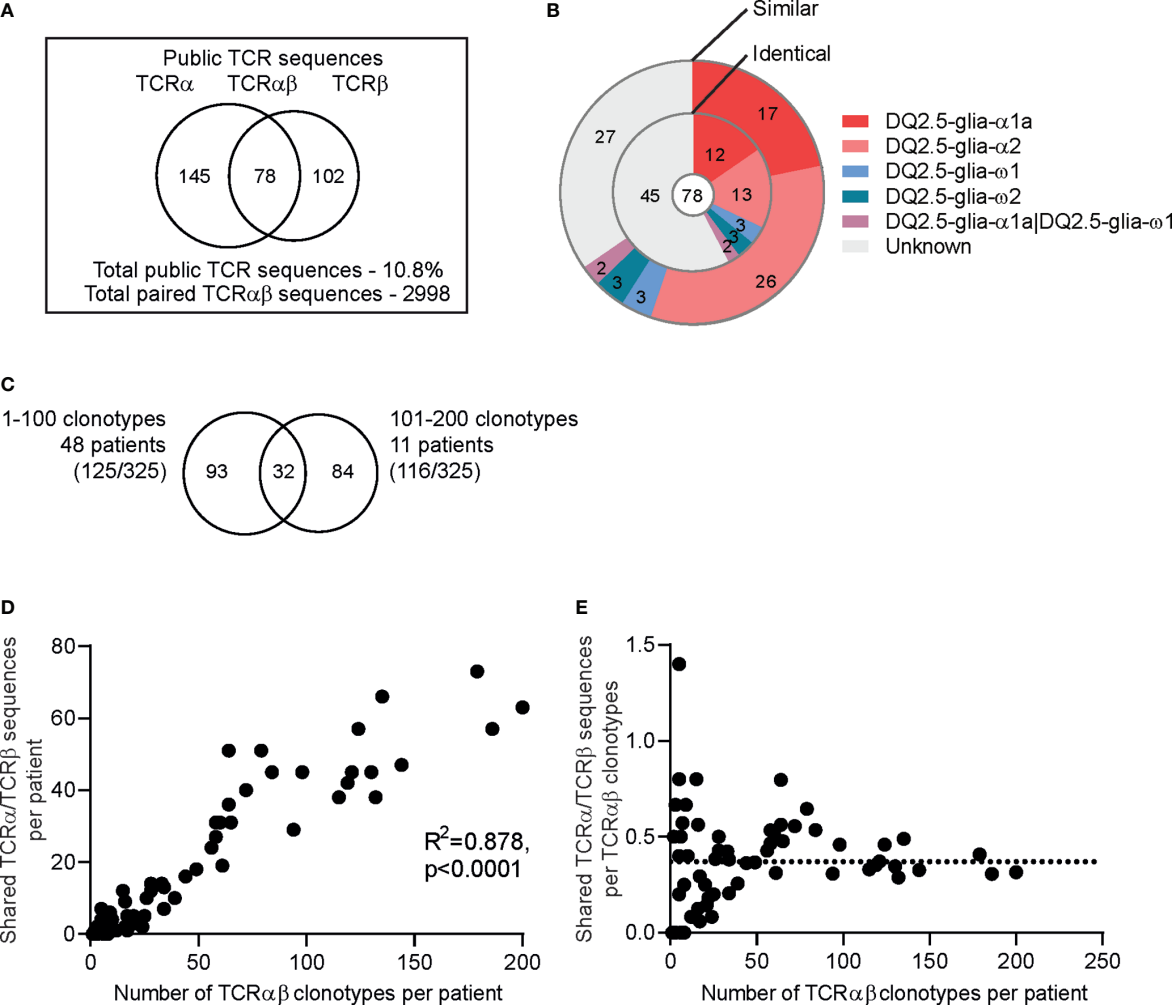
Public TCR sequences among gluten-specific T cells. **(A)** The number of public TCRs defined as identical TCRα, TCRβ or paired TCRαβ amino acid sequences observed at least in two individuals in a dataset of total 2996 gluten-specific TCRαβ sequences from 63 patients. **(B)** Doughnut chart showing the epitope specificity of the 78 public paired TCRαβ sequences. The inner circle displays the paired TCRαβ sequences that are identical to the TCRαβ sequences of TCRs with known epitope specificity while the outer circle displays the paired TCRαβ sequences that have similar (1 amino acid difference) TCRβ or TCRαβ sequences. **(C)** Plot showing the number of shared TCRα/TCRβ sequences and the number of TCRαβ clonotypes in each patient. **(D)** Intra-comparison of the shared TCRα/TCRβ sequences between patients with 1 to 100 TCRαβ clonotypes (n=47) and 101-200 TCRαβ clonotypes (n=11). Total number of shared sequences within the groups are shown in parentheses. **(E)** The number of shared TCRα/TCRβ sequences per TCRαβ clonotypes in each patient. The stippled line indicates the median value.

In order to identify TCR sequences that are shared across individuals (i.e. public sequences) among the gluten-specific T cells, we analyzed TCR sequences derived from T cells of blood, gut biopsies, as well as *in vitro* cultured T-cell lines and T-cell clones obtained from 63 CeD patients. Collecting all sequences from these 63 CeD patients, we obtained 2996 unique paired TCRαβ sequences at amino acid level from a total of 3122 clonotypes (3109 were unique at the nucleotide level). The 2996 sequences were used to analyze for public TCRs. Shared TCRα and TCRβ sequences were found in 59 out of 63 patients. The four remaining patients contained less than three TCRαβ clonotypes each making it less likely to detect public TCR sequences in these patients. We identified 325 TCRα or TCRβ sequences that were shared between 2 to 23 CeD patients (**Figure 3A**). Of the 325 public TCR sequences, 145, 102 and 78 were TCRα, TCRβ and paired TCRαβ sequences, respectively. Of the paired TCRαβ sequences, 42% were identical and 65% were highly similar to the TCRαβ clonotypes with known epitope specificity (**Figure 3B**, **Supplementary Table 7**).

We then assessed the contribution of the public TCRα and TCRβ sequences in the gluten-specific TCR repertoire of the individual patients. We found a higher number of shared TCRα or TCRα sequences in patients with higher number of TCRαβ clonotypes (**Figure 3C**). In order to investigate if the patients with higher number of TCRαβ clonotypes mostly contribute the public TCR sequences, we divided the patients into two groups where the first group contained patients with less than 100 clonotypes (1-100) and the second group contained those with more than 100 clonotypes (101-200). These two groups constituted almost equal number of clonotypes, but differed in number of patients which gave rise to two sets representing many patients-few clonotypes (47 patients, 1529 clonotypes) and few patients-many clonotypes (11 patients, 1585 clonotypes) (**Figure 3D**). Notably, we found that both groups contributed almost equally to the public TCR sequence pool. Further, we observed that the number of shared TCRα or TCRβ sequences per TCRαβ clonotypes in a given CeD patient appeared to be stable around 30-40% (median 37%) regardless of the number of gluten-specific clonotypes in the patient (**Figure 3E**).

### R-Motif Dominates the Public T-Cell Response to Gluten

**Figure 4:**
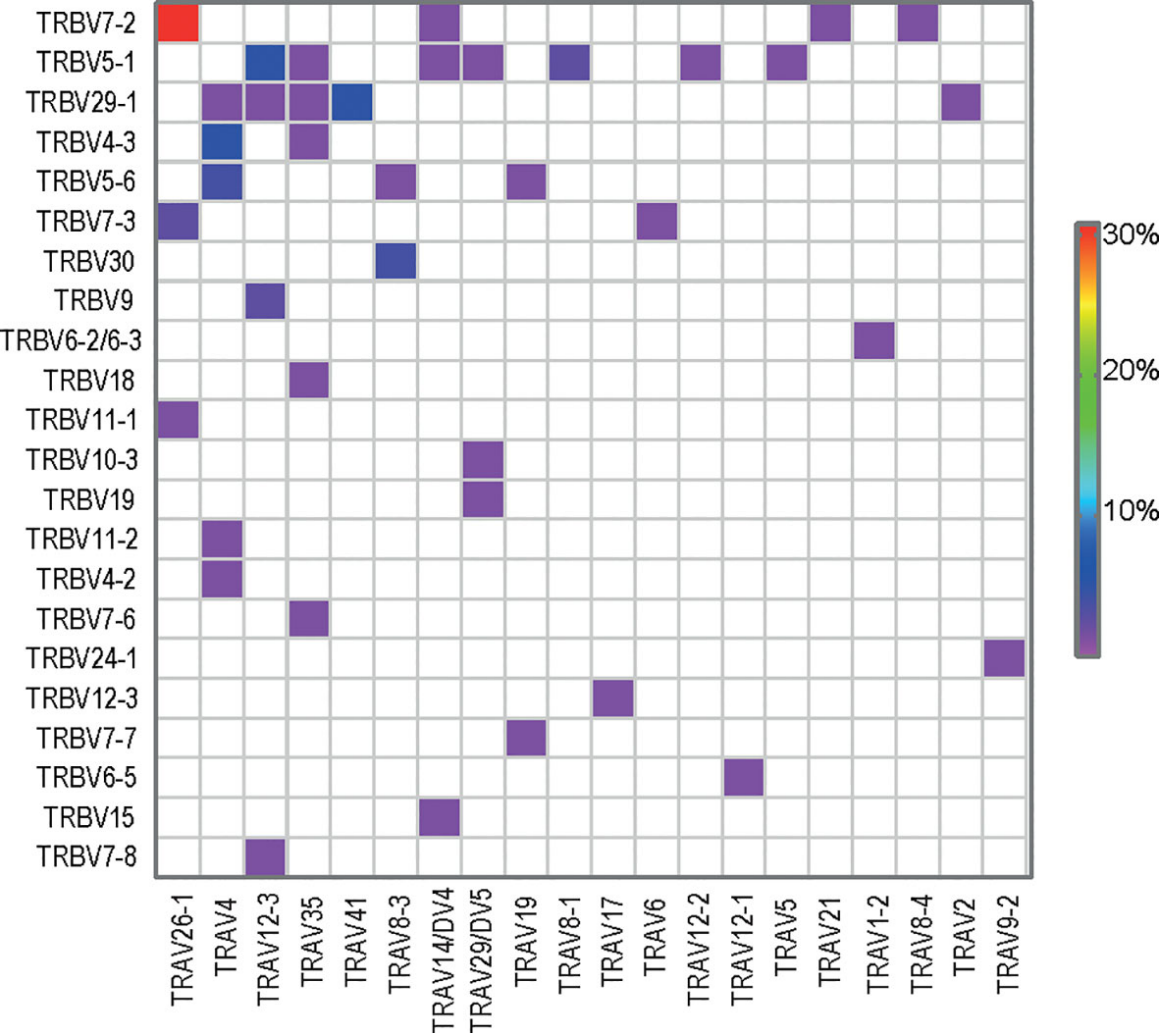
TRAV : TRBV gene usage in public paired TCRαβ sequences (n=78).

Upon analysis of the public paired TCRαβ sequences (n = 78), TRAV26-1:TRBV7-2 was the most dominant TRAV : TRBV pair (**Figure 4**). Further, the TCRβ sequences expressing the TRAV26-1:TRBV7-2/3 with R-motif were also the most prominent amongst the paired TCRαβ sequences (23%). When looking only into the collection of gluten-specific TCCs, 50% of the DQ2.5-glia-α2 specific TCCs (56/111) used TRBV7-2 and 30% also expressed the TRAV26-1 gene with the R-motif (33/111). Strikingly, we found T-cell clonotypes carrying the TRAV26-1:TRBV7-2 TCR with R-motif in all 41 patients where DQ2.5-glia-α2 tetramer was used. To sum up, the public T-cell response against wheat gluten in CeD is characterized by preferential expression of the TRAV26-1:TRBV7-2 and dominated by the use of the R-motif sequences.

### Public T-Cell Receptor Sequences Exhibit Higher Generation Probabilities

In order to investigate what makes a TCR sequence public, we compared the TCR sequences shared in more than two individuals (public) with the TCR sequences observed only in one individual (private) in our dataset.

Assuming that the sharing of TCR sequences across patients is most likely influenced by a higher immune receptor generation probability (IRGP), we compared the mean IRGP values for private and public sequences. We observed that public TCR sequences indeed had significantly higher IRGPs than private ones (**Table 3**). This applied both to individual chains and paired receptors. Given the below-average CDR3 length of R-motif sequences (11 versus ~12.5 amino acids) and its prominent representation amongst public TCRβ sequences, we additionally created TCRβ datasets without R-motif sequences. As expected, the mean IRGPs of public TCRβs still remained significantly higher than private TCRβ sequences in the R-motif-free TCRβ dataset.

**Table 3:** The mean IRGP values for public and private sequences, with the number of sequences given in parentheses.

Sample	Amino acid sequences			Nucleotide sequences		
	private: mean (n)	public: mean (n)	p-value (U)	private: mean (n)	public: mean (n)	p-value (U)
TCRα	3.18e-08 (2230)	5.96e-08 (182)	9.77E-04 (174966)	3.99e-09 (2475)	3.61e-08 (123)	5.82E-06 (116660.5)
TCRβ	3.01e-08 (2182)	8.35e-08 (149)	3.15E-34 (66042.5)	1.04e-09 (2615)	6.94e-09 (73)	4.42E-27 (25379.5)
w/o R-motif	3.01e-08 (2182)	4.73e-08 (115)	1.38E-20 (61494.5)	1.04e-09 (2615)	2.44e-09 (29)	6.59E-06 (20103)
TCRα:TCRβ	1.10e-15 (2600)	8.35e-15 (72)	5.68E-08 (59372)	1.01e-17 (2758)	3.12e-16 (11)	1.73E-03 (7441)

For each sample, the p-value and the U statistic for the one-sided Mann Whitney U test comparing the samples is given.

## Discussion

Unlike several other autoimmune disorders, the antigen (dietary gluten) driving the T-cell response is well established in CeD. Hence, lessons learned from CeD on features of the disease driving gluten-specific TCRs are likely applicable to other autoimmune disorders.

The features of the TCR repertoire against the individual immunodominant HLA-DQ2.5 restricted gluten epitopes have been studied previously (5, 7, 9, 11, 12). However, no reports on public TCR motifs used by T cells specific for other immunodominant HLA-DQ2.5 restricted gluten epitopes exist apart from the CDR3β (R-motif) and CDR3α (NDYKLS-motif) used by DQ2.5-glia-α2-specific TCRs (5, 7, 9, 11). In order to comprehensively search for potential new motifs, we performed hierarchical clustering of a large dataset of CDR3α and CDR3β sequences obtained from 2750 gluten-specific TCRαβ clonotypes from 34 CeD patients. While the R-motif cluster stands out as the most dominant motif, several new CDR3α, CDR3β and CDR3α:CDR3β motifs were identified. Strikingly, more than half of the new paired CDR3α:CDR3β motifs could be linked to one or two of the four epitope specificities by searching among TCRαβ clonotypes of epitope-specific *in vitro* cultured TCCs and T cells isolated with single HLA-DQ2.5:gluten tetramers, resulting in novel TCR motifs specific to DQ2.5-glia-α1a, DQ2.5-glia-α2, DQ2.5-glia-ω1, DQ2.5-glia-ω2 and DQ2.5-glia-α1a/DQ2.5-glia-ω1. Although we cannot rule out that each such motif may encompass TCRs reactive to further epitopes among these four, the strictness of the motifs makes us believe that the motifs mostly capture specificities to a single of these four epitopes. Analysis of whether these motifs are unique to CeD patients and can serve as proxies for the disease will have to be tested in series of samples from CeD patients and non-affected subjects.

Given the low frequency of gluten-specific T cells in blood and gut, we mostly used a pool of tetramers to maximize the yield of isolated gluten-specific T cells. While this approach had the limitation that it did not allow us to directly assign TCR usage with specificity, it was advantageous for the comparison of frequencies of TCR-motifs across epitopes.

The biased expression of TRAV26-1 and TRBV7-2 genes as well as the paired receptor resulting from these V genes emerged as the most prominent feature when analyzing the gene usage frequency of the gluten-specific T cells isolated using a cocktail of four immunodominant epitopes. V-gene bias has been proposed to be a consequence of several factors such as biases in somatic recombination, thymic selection, antigen-driven selection and structural features in TCR and peptide:MHC interaction (26–28). In line with this hypothesis, we speculate that the biased usage of TRAV26-1/TRBV7-2 TCR in T-cell response against gluten in CeD is the consequence of V-gene bias acting on numerous levels. The theory of antigen-driven selection bias suggests that the TCRs with a best fit for a peptide:MHC complex has an advantage over less-fit TCRs in the naive T-cell repertoire resulting in a biased repertoire (27). In this context, it is notable that the TCRs expressing TRAV26-1/TRBV7-2 with R-motif have shown higher affinity to the HLA-DQ2.5:DQ2.5-glia-α2 complex compared to the TRAV26-1/TRBV7-2 TCR with the extended R motif (8). Furthermore, the arginine that gives rise to the R-motif was shown to exert a key role in the interaction with HLA-DQ2.5:DQ2.5-glia-α2 complex (8). This indicates that the TRAV26-1/TRBV7-2 with R-motif due to its higher affinity to the peptide:MHC complex and crucial interactions might have an increased chances of selection during immune response resulting in increased prevalence in the subsequent effector pool.

The proportion of gluten-specific T-cell clonotypes that were public seemed to be around 30-40%, regardless of the number of gluten-specific TCRs we have acquired from each CeD patient. This indicates that these public T-cell receptors are central in the T-cell response against gluten. It has been proposed that the TCR sequences that are closer to the germline sequences and that could be easily generated are potential candidates for such public TCRs (26, 28). In agreement with this hypothesis, we observed that the public gluten-specific TCRs in our dataset had fewer N insertions in the CDR3 and higher generation probabilities. On scrutiny of the CDR3β sequence with the R-motif, the CDR3β sequence is relatively short and closely matches the V, D and J gene germline sequences. This motif also contains a conserved non-germline arginine at the fifth position, which has crucial role in the interaction between TCR and the DQ2.5-glia-α2 presented by HLA-DQ2.5 (8). However, the arginine in the R-motifs observed between individuals and within the same individual has shown to be encoded by different recombination events (nucleotides) (5, 16). As arginine is encoded by six codons, this increased variety of ways in which the R-motif amino-acid sequence can be made, thus creating a higher chance for TCR sharing across individuals. Instances of this phenomenon known as convergent recombination contributing to public T-cell response are also found outside CeD (28). Therefore, conceivably TCRs with the R-motif become dominating public TCRs due to the cumulative effect of utilizing the preferentially expressed V gene pair with near-germline CDR3 regions and the convergent recombination giving conserved non-germline arginine residues.

There are several examples in the literature on universal public motifs in human disorders (29–32), but it is unclear how often one may find strong public TCR motifs for any given specificity. The fact that we did not observe any other prominent motifs apart from the R-motifs despite employing HLA-DQ2.5:gluten tetramers covering four immunodominant gluten epitopes, suggests that the formation of dominant public TCR motifs does not take place for all peptide-MHC specificities. It is notable that no dominant public TCR motifs was observed for the DQ2.5-glia-ω2 epitope, an epitope that is highly homologous to the DQ2.5-glia-α2 epitope. Future studies should address to which extent public TCRs constitute the response to various antigens.

Looking for the presence of the CDR3 motifs identified in our study in the VDJdb and McPAS-TCR repositories, we found no matches for paired CDR3α:CDR3β motifs and very few matches for the CDR3α and CDR3β motifs indicating disease specificity of these TCR motifs. Potentially such information can be utilized in a celiac disease diagnostic test. Our result suggests that combined TCRα and TCRβ information will provide a better prediction. This notion of importance of both TCR chains is supported by the functional data demonstrating that T-cell reactivity in DQ2.5-glia-α2 specific TCRs using TRBV7-2 is lost upon substitution of TRAV26-1 with TRAV26-2 (33). In a recent study, Yao et al. analyzed for presence of TCR sequences of gluten-specific T cells in gut biopsies of 7 CeD patients and 8 disease controls, and concluded that correct disease status could be assigned based on presence of TCRs sequences in 13 out of 15 donors (34). While these results are encouraging for the prospect of a diagnostic test, further analysis is required to conclude how frequent the identified TCR CDR3 motifs are in healthy subjects. As there is a non-negligible number (about 1%) of undiagnosed celiacs in the general population (35), in future studies it will be important to accurately ascertain disease status of the disease controls.

Taken together, in this study we have demonstrated that the gluten-specific T-cell response is composed of one very dominant CDR3 motif used by DQ2.5-glia-α2-specific TCRs and several less dominant motifs used by TCRs specific for other dominant gluten-derived T-cell epitopes. Together, these CDR3 motifs are part of a diverse TCR repertoire employed by gluten-specific CD4+ T cells in CeD. Therefore, these public TCR sequences and conserved CDR3 motifs can potentially be exploited as diagnostic markers of CeD.

## Data Availability Statement

The datasets presented in this study can be found in online repositories. The names of the repository/repositories and accession number(s) can be found in the article/**Supplementary Material**.

## Ethics Statement

The studies involving human participants were reviewed and approved by Regional Committees for Medical and Health Research Ethics, Norway. Written informed consent to participate in this study was provided by the participants or the participants’ legal guardian/next of kin.

## Author Contributions

SD-K and LR isolated T cells and sequenced their receptors, performed data analysis and wrote the manuscript. RN performed data analysis and wrote the manuscript. AC isolated T cells. KL organized collection of CeD patient material. GS supervised the project and critically revised the manuscript. S-WQ isolated T cells, supervised the project and critically revised the manuscript. LS supervised the project, arranged the necessary funding and wrote the manuscript. All authors contributed to the article and approved the submitted version.

## Funding

This work has been supported by grants from Stiftelsen Kristian Gerhard Jebsen (project SKGJ-MED-017), from the Research Council of Norway (project 179573/V40 through the Centre of Excellence funding scheme and project 233885) and from the South-Eastern Norway Regional Health Authority (projects 2011050, 2013046, 2015009 and 2018068).

## Conflict of Interest

SD-K, LR, RN, AC, KL, S-WQ, GS and LS are holders of a patent application entitled "Method of diagnosing celiac disease" (US20210010077A1) on the use of the gluten-specific T cell receptor sequences described in the current work for diagnosis of celiac disease.
