# Supplementary material for: Comprehensive Analysis of CDR3 Sequences in Gluten-Specific T-Cell Receptors Reveals a Dominant R-Motif and Several New Minor Motifs
Source: Front Immunol. 2021 Apr 13;12:639672. doi: 10.3389/fimmu.2021.639672 (PMC8076556; doi:10.3389/fimmu.2021.639672)
Supplement: Supplementary file 1 [file DataSheet_1.pdf]

Supplementary Figure 1

Gating strategy of tetramer stained cells

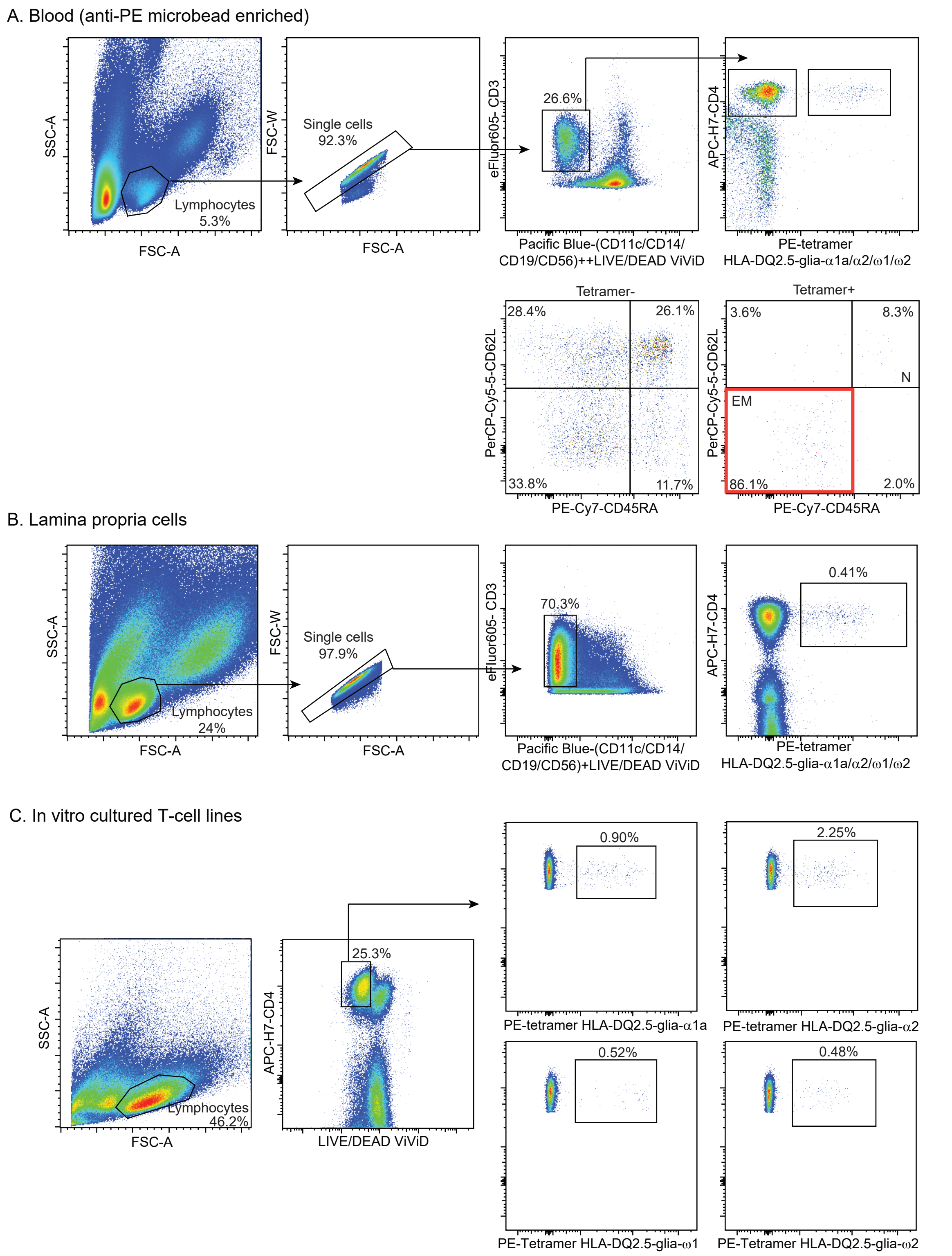

Supplementary Figure 1. Representative plots showing the gating strategy used to isolate HLA-DQ2.5:gluten tetramer binding CD4+ T cells from (A) blood, (B) gut biopsies and (C) T-cell lines. (A) Live cells within the singlet lymphocyte population were gated to obtain CD3+, CD11c−, CD14−, CD15−, CD19−, CD56−, CD4+, CD45RA−, CD62L−, (integrin β7+), and HLA-DQ2.5:gluten tetramer+ cells from PBMCs. (B) Live single lymphocytes that were CD3+, CD11c−, CD14−, CD15−, CD19−, CD56−, CD4+, CD8−, HLA-DQ2.5:gluten tetramer+ were isolated from single-cell suspension of the gut biopsies. (C) Live single lymphocyte cells that were CD3+, CD4+, HLA-DQ2.5:gluten tetramer+ were sorted from T-cell lines.

Supplementary Figure 2

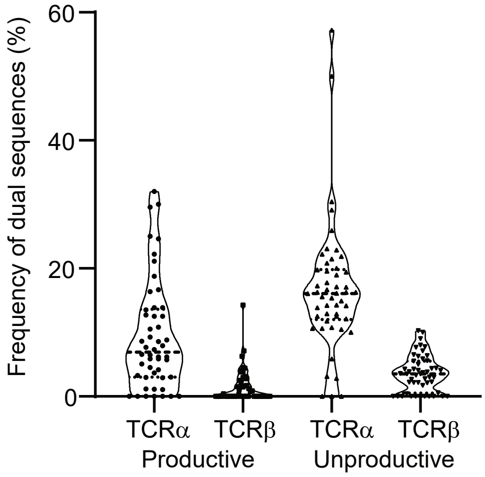

Supplementary Figure 2. Frequency of dual productive and unproductive TCRα and TCRβ sequences in each patient is shown in the violin plot.

Supplementary Figure 3

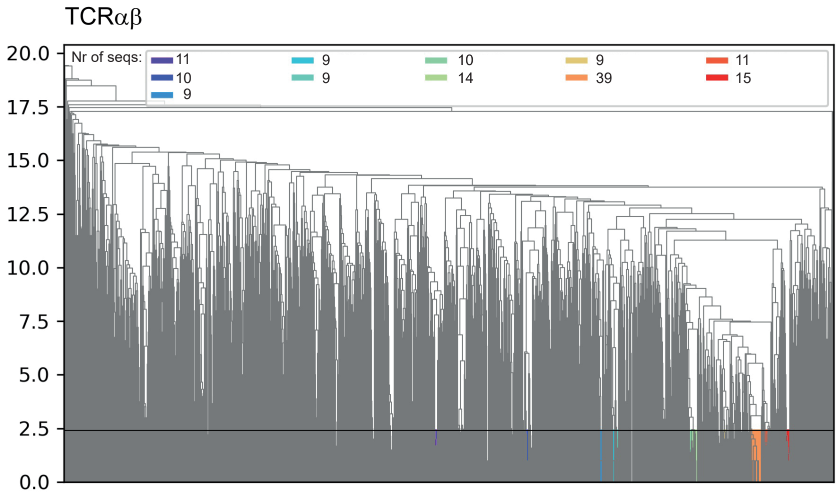

Supplementary Figure 3. Hierarchical clustering of paired CDR3α:CDR3β sequences. The y-axis indicates the Levenshtein distance and the horizontal line shows distance that was used to cut the dendogram tree to generate the clusters. The clusters are colored and the respective number of sequences in each cluster are indicated in the legend.

Supplementary Figure 4

A

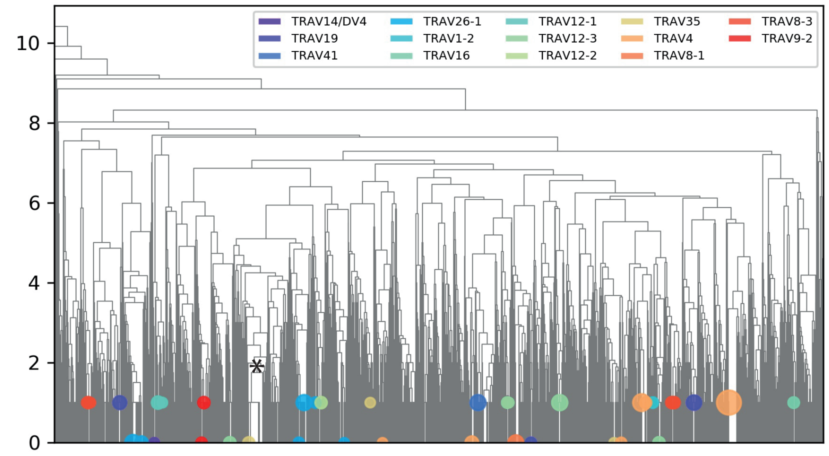

B

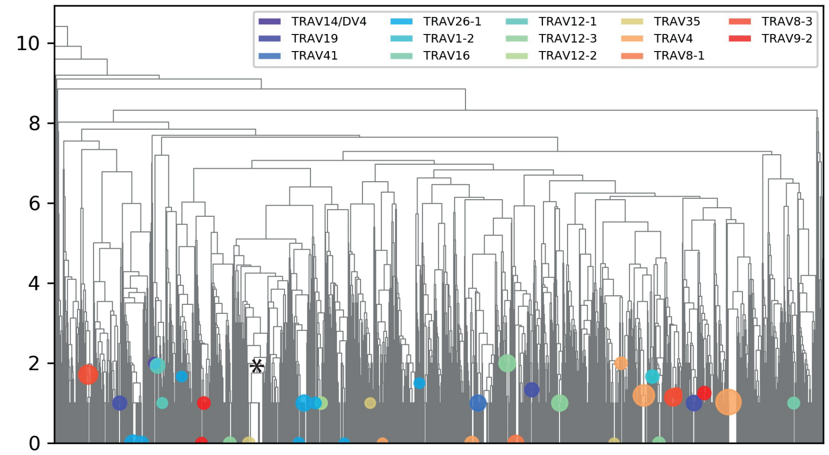

C

Id	TRAV	CDR3α motif
2429*	TRAV4	LVGDDTGFQKLV
2104*	TRAV4	LVGGSGGYNKLI
116	TRAV8-3	AVGTDRGSTLGRLY
878	TRAV26-1	IVLNARLM
2204	TRAV8-3	AVGAAEYGNKLV
267	TRAV26-1	IVYGGSQGNLI
1612	TRAV12-3	AMSELPGGSNYKLT
1649	TRAV8-1	AVNARNAGNMLT
1799	TRAV12-3	AMTDYGNNRLA
1509	TRAV41	AVESGSNYQLI
363	TRAV12-1	VVNLASSASKII
227	TRAV19	ALSEAFGAGGTSYGKLT
937	TRAV12-2	AVSNRDDKII
2278	TRAV19	ALSEGGNAGNMLT
1487	TRAV4	LVGEGDSNYQLI
291	TRAV26-1	IGNYGGSQGNLI
1698	TRAV19	ALSEGGNAGNMLT
2210	TRAV8-3	AVGVSEYGNKLV
442	TRAV26-1	IVINGQNFV
2313	TRAV9-2	ALSDPTGTASKLT
609	TRAV12-3	AMSAGTGNQFY
517	TRAV9-2	ALSEGNFNKFY
509	TRAV9-2	ALSEYNFNKFY
350	TRAV14	AMREGRYSSASKII
2149*	TRAV12-3	AMIEAAGNKLT
2017	TRAV4	LVGDGDGGATNKLI
860	TRAV26-1	IXFNARLM
1983	TRAV35	AGNYGGATNKLI
925	TRAV26-1	IVRVVGDDKII
1111	TRAV35	AGQPGSSNTGKLI
2129	TRAV1-2	AVRAVESGGYNKLI
1016	TRAV26-1	ICYNNNDMR
1293	TRAV26-1	IVASGSARQLT
1166	TRAV4	LVGGDNQGGKLI
681	TRAV35	AGNDYKLS
2639	TRAV16	ALNSGGYQKVT
372	TRAV12-1	VVNLYSSASKII

Supplementary Figure 4. New CDR3α motif clusters with a maximal Levenshtein distance of (A) 1.0 and (B) 2.0 derived from hierarchical clustering of CDR3α sequences. Parent nodes of potential CDR3α motifs are indicated. Circle sizes are proportional to the number of comprised clonotypes. All the clonotypes belonging to the same parent node are utilizing identical V genes as indicated by the color of the parent node. Asterisk shows the cluster containing the NDYKLS-motif. (C) All the CDR3α motifs at Levenshtein distance 2.0 with the sequence logo. Previously described motifs are indicated by asterisk.

Supplementary Figure 5

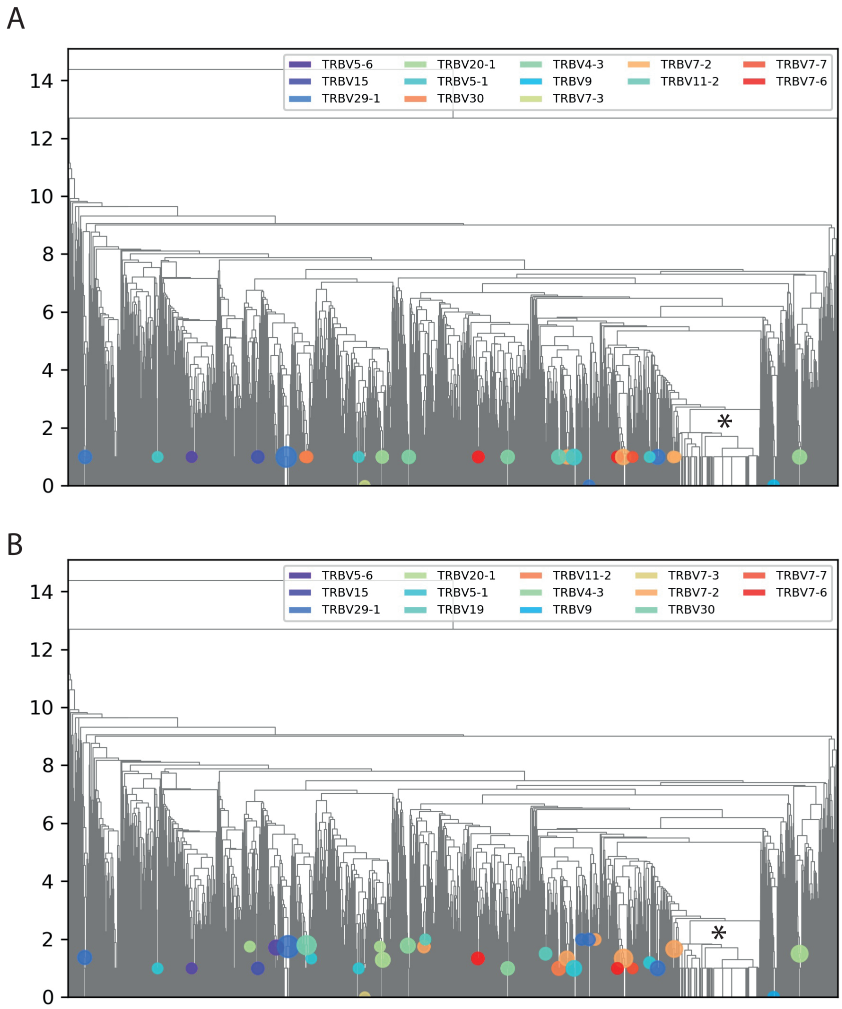

C

Id	TRBV	CDR3β motif
1083	TRBV20-1	SASKQLNTEAF
1963*	TRBV7-2	ASSLRAGGADTQY
759*	TRBV29-1	SVGQGGTGELF
823	TRBV30	AWSVTGWDTGELF
716	TRBV5-6	ASSFGVTGELF
1092	TRBV20-1	SASRQVNTEAF
2128	TRBV7-2	ASSLRTGDTQY
1781	TRBV5-1	ASSFDGETQY
1728	TRBV11-2	ASSSTAQETQY
2605	TRBV20-1	SARRQADQPQH
1757	TRBV7-2	ASSLVGWETQY
1252	TRBV7-2	ASSIRTSGGHEQF
1192	TRBV4-3	ASSQGLAGRQETQY
2078*	TRBV29-1	SVGAVSTDTQY
50	TRBV29-1	SVGGSGANVLT
655	TRBV15	ATSRAGGGGEKLF
1680	TRBV19	ASSIGTSGETQY
1544	TRBV4-3	ASSQGSGGNEQF
1441	TRBV7-6	ASSFGSYNEQF
2493	TRBV9	ASSVGGGAGDTQY
1832	TRBV29-1	SAGQGETQY
1935	TRBV7-6	ASSLAAAGGTDTQY
2049	TRBV5-1	ASSLGQPSTDTQY
1808	TRBV29-1	SVATGWSETQY
1859	TRBV7-2	ASSLRPGGETQY
838	TRBV5-1	ASSLGGPNTGELF
1985	TRBV7-7	ASSALAGGDTQY
1256	TRBV19	ASSIRTGGISEQY
1029	TRBV7-3	ASSQGQDTEAF
628	TRBV20-1	SASPGDEKLF
403	TRBV5-6	ASSLGRSYGYT
1010	TRBV5-1	ASSLVAWDTEAF

Supplementary Figure 5. New CDR3β motif clusters with a maximal Levenshtein distance of (A) 1.0 and (B) 2.0 derived from hierarchical clustering of CDR3β sequences. Parent nodes of potential CDR3β motifs are indicated. Circle sizes are proportional to the number of comprised clonotypes. All the clonotypes belonging to the same parent node are utilizing identical V genes as indicated by the color of the parent node. Asterisk shows the cluster containing the R-motif. (C) All the CDR3β motifs at Levenshtein distance 2.0 with the sequence logo. Previously described motifs are indicated by asterisk.

Supplementary Figure 6

A

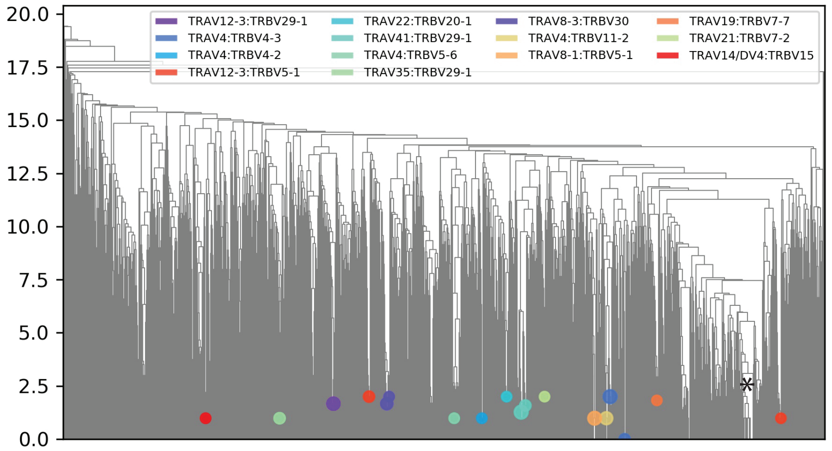

B

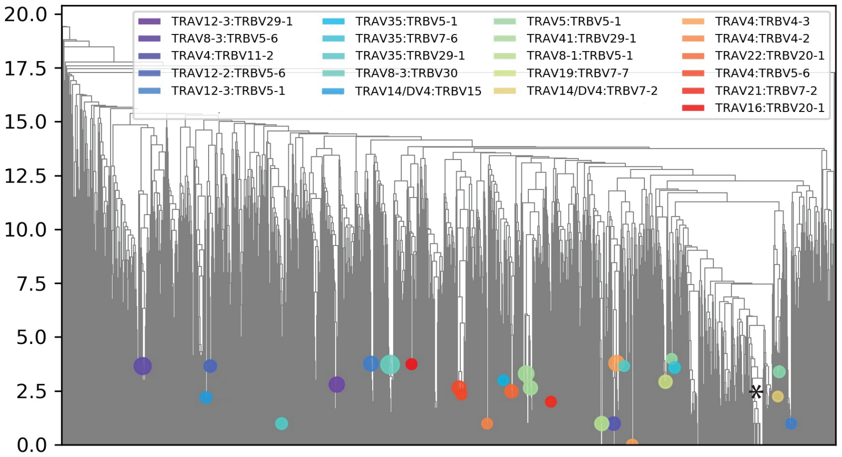

C

Id	TRAV	CDR3α motif	TRBV	CDR3β motif
1383	TRAV4	LVGDDTGFQKLV	TRBV5-6	ASSBRQGYGYT
1131	TRAV8-3	AVGAAEYGNKLV	TRBV30	AWSYTGWDTGELF
270	TRAV8-3	AVGTDRGSTLGRLY	TRBV5-6	ASSFSVTGELF
1933	TRAV4	LVGGSGGYNKLI	TRBV4-3	ASSQGLAGRQETQY
1631	TRAV41	AVEAGSNYQLI	TRBV29-1	SVGGSGANVLT
1066	TRAV12-3	AMTDYGNNRLA	TRBV5-1	ASSLAGPNTGELF
503	TRAV12-2	AVSNRDDKII	TRBV5-6	ASSLRTEYSGNTIY
1872	TRAV8-1	AVNARNAGNMLT	TRBV5-1	ASSFDGETQY
2094	TRAV19	ALSGAGANSKLT	TRBV7-7	ASSELAGGDTQY
1620	TRAV41	AVEGGSNYKLT	TRBV29-1	SVGAGGTGELF
932	TRAV12-3	AMSAGTGNQFY	TRBV29-1	SVGAVSTDTQY
1555	TRAV22	AVEHQGAQKLV	TRBV20-1	SASRQVNTEAF
1379	TRAV4	LVGDDTGFQKLV	TRBV5-6	ASSLGRSYGYT
1473	TRAV4	LVGGDNQGGKLI	TRBV4-2	ASSQRQGGNTIY
488	TRAV14	AMREGRYSSASKII	TRBV15	ATSRAGGGGEKLF
741	TRAV35	AGQEGSSNTGKLI	TRBV29-1	SAGQGETQY
2133	TRAV35	AGPEHYNTDKLI	TRBV7-6	ASSLAAAGGTDTQY
2506	TRAV26-1	IVYNTGFQKLV	TRBV7-2	ASSLRAGGADTQY
1956	TRAV35	AGNYGGATNKLI	TRBV29-1	SVDKGGDTETQY
1693	TRAV21	AVPSGAGSYQLT	TRBV7-2	ASSLVGWETQY
1986	TRAV4	LVGDGDGGATNKLI	TRBV4-3	ASSQGSGGNEQF
2536	TRAV12-3	AMIEAAGNKLT	TRBV5-1	ASSFGGGAGDTQY
1919	TRAV4	LVGGSGGYNKLI	TRBV11-2	ASSSTAQETQY
1208	TRAV16	ALNSGGYQKVT	TRBV20-1	SASSLASQNEQF
1525	TRAV35	AGQL[illegible]GGATNKLI	TRBV5-1	ASSLVAWDTEAF
2123	TRAV5	AESHTDKLI	TRBV5-1	ASSFGQPSTDTQY
2497	TRAV14	AMNTGGFKTI	TRBV7-2	ASSLRAGGADTQY

Supplementary Figure 6. New paired CDR3α:CDR3β motif clusters with a maximal Levenshtein distance of 2.0 (A) and 4.0 (B) derived from hierarchical clustering of paired CDR3α:CDR3β sequences. Parent nodes of potential CDR3α:CDR3β motifs are indicated. Circle sizes are proportional to the number of comprised clonotypes; all clonotypes belonging to the same parent node are utilizing identical paired TRAV and TRBV genes as indicated by the color of the parent node. Asterisk shows the cluster containing the R-motif paired to the NDYKLS-motif. (C) All the CDR3α:CDR3β motifs at Levenshtein distance 2.0 depicted with sequence logos.
